# A Novel Form of Neuregulin 1 Type III Caused by N-Terminal Processing

**DOI:** 10.3390/biom13121756

**Published:** 2023-12-07

**Authors:** Yukai Wang, Yu Zhang, Yingxing Wang, Hong Chen, Liangjing Pan, Xufeng Liao, Shunqi Wang

**Affiliations:** 1 School of Life Sciences, Nanchang University, Nanchang 330031, China; 2 Institute of Biomedical Innovation, Jiangxi Medical College, Nanchang University, Nanchang 330031, China; 3 School of Basic Medical Sciences, Jiangxi Medical College, Nanchang University, Nanchang 330031, China

**Keywords:** Nrg1 type III, transmembrane domain, processing

## Abstract

Nrg1 (Neuregulin 1) type III, a susceptible gene of schizophrenia, exhibits a critical role in the central nervous system and is essential at each stage of Schwann’s cell development. Nrg1 type III comprises double-pass transmembrane domains, with the N-terminal and C-terminal localizing inside the cells. The N-terminal transmembrane helix partially overlaps with the cysteine-rich domain (CRD). In this study, Nrg1 type III constructs with different tags were transformed into cultured cells to verify whether CRD destroyed the transmembrane helix formation. We took advantage of immunofluorescent and immunoprecipitation assays on whole cells and analyzed the N-terminal distribution. Astonishingly, we found that a novel form of Nrg1 type III, about 10% of Nrg1 type III, omitted the N-terminal transmembrane helix, with the N-terminal positioning outside the membrane. The results indicated that the novel single-pass transmembrane status was a minor form of Nrg1 type III caused by N-terminal processing, while the major form was a double-pass transmembrane status.

## 1. Introduction

Nrg1 (Neuregulin 1) is one of the widely studied susceptible genes for schizophrenia [1,2,3,4]. Nrg1 spans 1.125 mega-bases comprising over 20 exons, and more than 30 splice isoforms are grouped into six types (Ⅰ–Ⅵ) [1,5]. All Nrg1 isoforms share a domain similar to the epidermal growth factor (EGF) in the extracellular and activate the tyrosine kinase function of the ErbB receptor [1]. NRG1 type III, a member of Nrg1, is not only the most prominent Nrg1 isoform in the sensory neurons of the dorsal root ganglia and motoneurons of the spinal cord but also the most highly expressed isoform in the brain [1,5].

Nrg1 type III is a critical signal that controls Schwann cell specification [6], migration [7], proliferation [8], apoptosis, and myelination [9] in developing nerves through multiple signaling pathways, such as PI3K/Akt, MAPK/Erk, and calcineurin/NFATc4 [10,11,12,13]. In cultured Schwann cells, Nrg1 type III is identified as the unique signal responsible for activating NF-κB, a critical factor for the cells to differentiate into the myelinating phenotype [6,14]. A low level of Nrg1 type III is required for axon ensheathment in the peripheral nervous system (PNS), whereas a high level triggers myelination independent of the axonal diameter [15]. Expression of Nrg1 type III can rescue the poorly ensheathed sensory neurons from Nrg1 type III deficient mice and can convert the ordinarily unmyelinated axons to myelination by enhancing PI3-kinase activity [11,12,16]. However, Nrg1 type III is dispensable in maintaining the myelin sheath in adult animals, since there is no effect on axon diameter and *g*-ratio in Nrg1 type III knockout adults [17].

Surprisingly, Nrg1 type III is prominently expressed in the dendrites in both the developing and adult central nervous system (CNS) but not detectable in the myelinated tracts or individual axons of the CNS [18]. Therefore, Nrg1 type III seems to play a role only in stimulating oligodendrocyte differentiation and the initiation of myelination [16]. However, a persistent high expression of Nrg1 type III from p14 (postnatal day 14) to p56 in mice indicates that it exhibits a continued role in the mature CNS [19]. Moreover, mice with Nrg1 type III overexpression exhibit sex-dependent behaviors in schizophrenia-associate tests or amyotrophic lateral sclerosis (ALS) models. Male mice with Nrg1 type III overexpression perform several schizophrenia-associate behaviors, such as contextual fear conditioning, social recognition memory deficits, and deficient sensorimotor gating [20,21]. However, Nrg1 type III overexpression in female mice alters the locomotor response to MK-801 without modifying other schizophrenia-associated behaviors [20]. Nrg1 type III can retain neuromuscular function, refine locomotor performance, amplify the surviving motor neuron number, and decrease glial reactivity in female mice with ALS, but male mice with ALS do not exhibit the benefits seen in the female mice [22,23].

High levels of Nrg1 type III mRNA or protein have been found in the brains of schizophrenia patients [16,24]. In addition, one research study has revealed that the haplotype Nrg1 causes a strong expression of Nrg1 type III in the brain, which may cause the pathogenesis of schizophrenia [2]. Conversely, recent research suggests that Nrg1 type III has a protective potential in schizophrenia pathogenesis [25]. Methamphetamine has been shown to augment the risk of schizophrenia and psychosis [26,27], and Nrg1 type III overexpression mice are somewhat less sensitive to the effects of methamphetamine, including prepulse inhibition and locomotion [25].

Most Nrg1 isoforms contain a single-pass transmembrane domain (TMD), whereas Nrg1 type III includes double-pass TMDs with the EGF domain in the extracellular loop [28]. Its ectodomain can be cleaved by three sheddases, namely, BACE1, ADAM10, and ADAM17, and the cleavages localize in the C-terminal to the EGF domain [29,30,31,32]. The N-terminal cytoplasmic region of Nrg1 type III harbors a unique cysteine-rich domain (CRD), which overlaps with the transmembrane helix in the N-terminal TMD [15], endowing it with the ability to act as a juxtracrine signal [6,16]. Therefore, the membrane topology indicates that Nrg1 type III is closely associated with the cellular membrane [28]. The N-terminal cytoplasmic domain of Nrg1 type III is considered as an intrinsically disordered structure with the potential to interact with metal ions in vitro [15].

In this study, Nrg1 type III constructs with different tags were transformed into cultured cells to verify whether the CRD destroyed the transmembrane helix formation. About 10% of Nrg1 type III omitted the N-terminal transmembrane helix, with the N-terminal positioning outside the membrane. The results indicated that the novel single-pass transmembrane status was a minor form of Nrg1 type III resulting from N-terminal processing, while the major form was of double-pass transmembrane status.

## 2. Methods

### 2.1. Cell Line and Culture Condition

HEK293 and N2a cells were kept in our lab, and the cells were cultured as previously described [33]. Both cell lines were maintained in DMEM (Dulbecco’s modified Eagle medium, Thermo Fisher Scientific, Waltham, MA, USA) supplemented with 10% FBS (Fetal bovine serum, Thermo Fisher Scientific) and 1× Penicillin–Streptomycin Solution (Thermo Fisher Scientific) in tissue culture dishes or culture plates with a poly-D-Lysine-coated coverslip [34], in a humidified incubator at 37 °C with an atmosphere of 95% air and 5% carbon dioxide.

To enhance Nrg1 type III secretion and outside distribution on the membrane [29], the cell culture was supplemented with ADAM inhibitor GM6001(100 nM, CC1010, Sigma Aldrich, St. Louis, MO, USA), BACE1 inhibitor Ⅳ (100 nM, S4562, Sigma Aldrich), and PMA (Phorbol 12-myristate 13-acetate; 100 ng/mL; S1819, Beyotime Biotechnology, Haimen, China).

### 2.2. Plasmids

The full length of Nrg1 type III (700 amino-acid residues) was cloned into the p3xFlag-Myc-24 construct as p3xFlag-Myc-Nrg1 type III, with Flag-tag in the N-terminal and Myc-tag in the C-terminal. HA-tag (amino-acid residues: YPYDVPDYA; nucleic-acid sequence: TACCCATACGATGTTCCAGATTACGCT) was inserted into amino-acid residue 233–234 of Nrg1 type III in the construct of p3xFlag-Myc-Nrg1 type III, following the instructions of the Quick-change Lightning Multi Site-Directed Mutagenesis Kit (Cat # 210514, Agilent Technologies, Santa Clara, CA, USA), namely, as p3xFlag-HA-Myc-Nrg1 type III.

### 2.3. Western Blotting (WB)

WB was performed as previously described, with minor modifications [35]. In brief, the cells were collected, and the proteins were extracted via RIPA (1.0% NP-40, 150 mm NaCl, 50 mm pH 8.0 Tris, 0.5% Na-deoxycholate, 0.1% SDS), supplemented with 1% PMSF (Phenyl-methane sulfonyl fluoride) and protease inhibitor cocktail (Cat. #5871, Cell Signaling Technology, Danvers, MA, USA) before use. After electrophoresis, samples were transferred to the PVDF membrane (Millipore, Saint Charles, MO, USA). The membrane was blocked using 5% skim milk for 2 h. We washed the membrane 3 times. Anti-Flag (Cat. # F1804, mouse, 1:2000, Sigma Aldrich) and anti-Myc (Cat. # SC-40, mouse, 1:1000, Santa Cruz Bicycles, Santa Cruz, CA, USA) primary antibody was added and incubated at 4 °C overnight. The HRP-labeled secondary antibody was added (Goat anti-Mouse IgG 31431, Thermo Fisher Scientific, 1:2000) to set at room temperature for 2 h and then washed 3 times. Immunoreacted bands were captured using an enhanced chemiluminescence system (BIO-RAD, Boulder, CO, USA).

### 2.4. Immunocytochemistry

**Figure 1:**
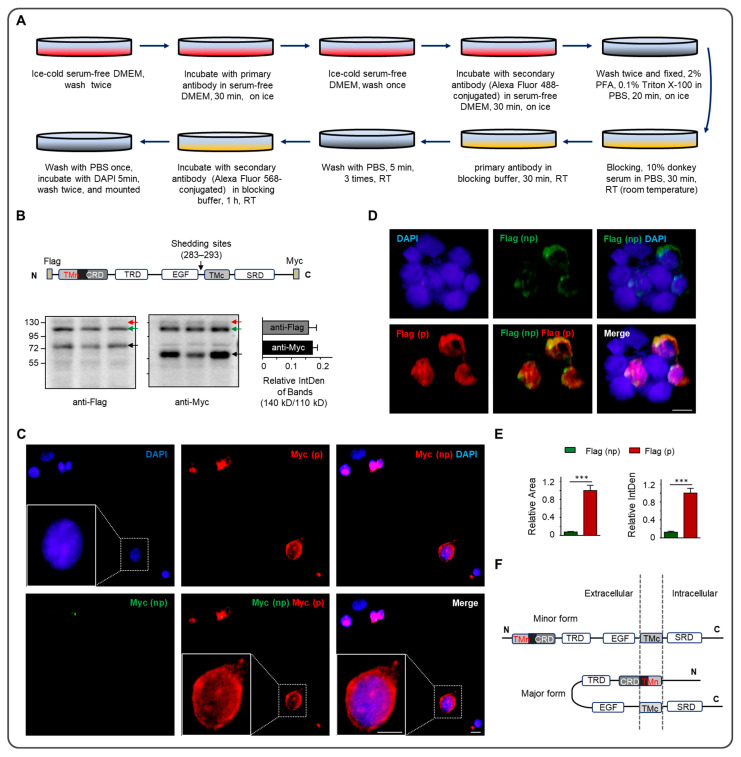
The N-terminal processing and localization of Nrg1 type III on the cell membrane. (**A**) The schematic procedure for the immunocytochemistry. (**B**) Upper panel: the schematic structure of the Nrg1 type III construct (p3xFlag-Myc-Nrg1 type III) with the insertion of the Flag and Myc tags in the N-terminal and C-terminal, separately. The black arrow shows the amino acid residues of shedding sites (ARSs) from three sheddases. Lower panel: the expression of the Nrg1 type III construct was verified through Western blotting via the two tags, and identical triple samples were uploaded in three lanes; moreover, the quantification of the relative bands’ abundance of 140 kD and 110 kD is shown in the lower left panel. The red and green arrows indicate the full length of Nrg1 type III, with different molecular weights of 140 kD (mature) and 110 kD (immature), and the black arrow shows the fragments (76 kD and 60 kD) after the cut in the ARSs. TMn is overlapped with CRD. TMn: transmembrane near the N-terminal; CRD: cysteine-rich domain; TRD: threonine-rich domain; EGF: epidermal growth factor (EGF)-like domain; TMc: transmembrane near the C-terminal; SRD: serine-rich domain. (**C**,**D**) Representative images of an N2a cell expressing the Nrg1 type III construct in the immunocytochemistry; (np) and (p) indicate staining before or after cell permeabilization. Anti-Myc (np) staining before the permeabilization of the cell was followed by staining with anti-Myc (p) (**C**). The large white rectangle area in panel C magnifies the small rectangle area. There were no less than three replicates for each experiment. Anti-Flag staining before and after permeabilization is shown in panel (**D**). (**E**) The relative staining size and IntDen (integrated density) from Flag (np) to Flag (p) were analyzed using software (ImageJ version 1.51w and GraphPad Prism 6). The values of the staining from permeabilization were normalized to 1. The value of the green column in the upper panel = 0.076 ± 0.011, and the green column in the lower panel = 0.127 ± 0.021; nonparametric statistical analysis via unpaired *t*-test, *** *p* < 0.001, *n* = 29. (**F**) The schematic model of the localization of Nrg1 type III on the cell membrane. The major form of Nrg1 type III was a double-pass transmembrane, and the minor configuration was a single-pass transmembrane (scale bar = 5 μm). (p3xFlag-Myc-Nrg1 type III) with Flag and Myc tags, separately, in the N-terminal and C-terminal were transfected into HEK293 cells (**A**). After the cleavage at the shedding sites (amino acid residue 283–293), 76 kD of the N-terminal with EGF domain and 60 kD fragment of the C-terminal were produced (shown by the black arrow in (**B**)) as previous reported [28,29,30]. Moreover, the mature form (140 kD) was less than the immature form (110 kD), whose relative abundance was no more than 0.2 based on the analysis of anti-Flag and anti-Myc blotting bands. Original Western Blotting Figures can be found in File S1.

**Figure 2:**
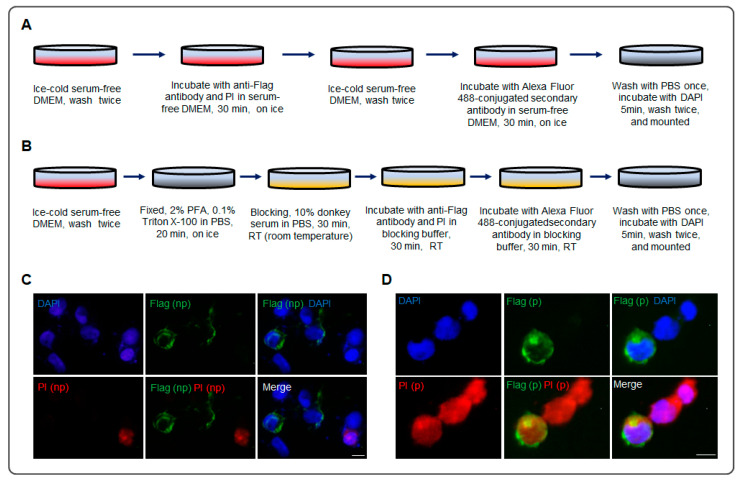
The N-terminal of Neuregulin 1 (Nrg1) type III localization on the cell membrane. (**A**,**B**) The schematic procedure for anti-Flag co-staining with PI before (**A**) or after (**B**) permeabilization simultaneously. (**C**,**D**) In the immunocytochemistry, representative images of HEK293 cells expressing the Nrg1 type III construct. Anti-Flag co-stained with propidium iodide (PI) before permeabilization (**A**) or after permeabilization (**B**). Nrg1 type III construct (p3xFlag-Myc-Nrg1 type III) was inserted with Flag and Myc tag at the N-terminal and C-terminal, separately, the same as the construct in Figure 1. (np) indicates the staining before cell permeabilization; (p) indicates the staining after cell permeabilization (scale bar = 5 μm).

**Figure 3:**
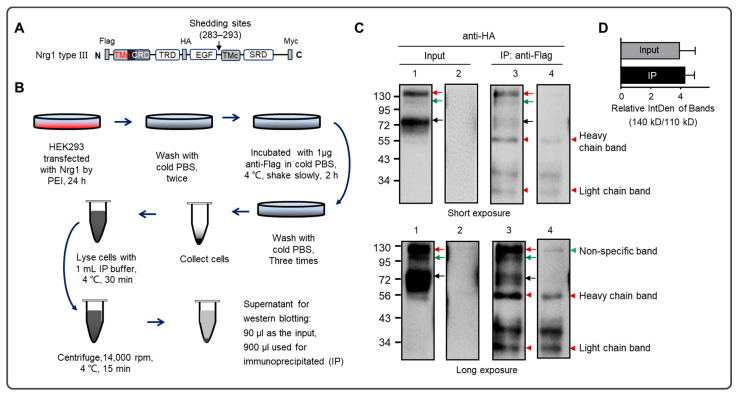
The anti-N-terminal antibody can isolate Nrg1 type III on the whole cell. (**A**) The schematic structure of the Nrg1 type III constructs (p3xFlag-HA-Myc-Nrg1 type III) was inserted with an HA tag in the middle of p3xFlag-Myc-Nrg1 type III, and the black arrow shows the amino acid residues of shedding sites (ARSs) by three sheddases. The construct was used in the immunoprecipitation (IP) assay of panels B and C. (**B**) The procedure for the IP assay on the whole-cell level and the samples were used in the Western blotting in panel C. After the first wash step, only cells that have outgrown and attached to the surface can be labeled via anti-Flag antibodies. After incubating with IP antibody (anti-Flag), the wash removed the unbound antibody. After lysing the cells, the IP antibody may change to bind the antigen from the double-pass transmembrane form of Nrg1 type III, including its full length and N-terminal fragment. The bands indicated by arrows (red, green, and black) in the IP lane of panel C were attributed to the interaction in incubating the IP antibody with the antigen on the surface of the cells. (**C**) The N-terminal of Nrg1 type III localization outside the cell was confirmed. The anti-N-terminal antibody (Flag tag) could immunoprecipitate specific Nrg1 type III; three replicates showed similar results. The inputs and IP were blotted with anti-HA antibodies; 1, 3, Nrg1 type III; 2, 4, the control construct is the empty vector without insertion at the multiple cloning site. The red and green arrows indicated the full length of Nrg1 type III, with different molecular weights of 140 kD (mature) and 110 kD (immature), and the black arrow showed the fragments (76 kD) after cutting in the ARSs. The green arrowhead indicated a nonspecific band after prolonged exposure. The red arrowhead showed the heavy- and light-chain bands, and the bands between the heavy- and light-chain bands were nonspecific bands. (**D**) The quantification of the relative bands’ abundances of the mature form (140 kD) and the immature form (110 kD) using Western blotting. Original Western Blotting Figures can be found in File S2.

Immunocytochemistry was performed according to the previous study [28,36], with minor modifications. After HEK293 (Figure 1 and Figure 2) or N2a (Figure 3) cells were seeded on the poly-D-Lysine-coated coverslip in the plate for 24 h, the construct of p3xFlag-Myc-Nrg1 type III was transfected into the cells. The following procedure is shown in Figure 1A. In brief, immunocytochemistry included incubating primary and secondary antibodies with the cells before and after permeabilization. Anti-Flag (Cat. # F1804, mouse, 1:1000, Sigma Aldrich) and anti-Myc (Cat. # SC-40, mouse, 1:1000, Santa Cruz Bicycles) antibodies were used. Propidium iodide (PI, 1:10,000) was co-stained with anti-Flag before (procedure shown in Figure 2A) or after (procedure shown in Figure 2B) permeabilization simultaneously. Alexa Fluor 488/568-conjugated goat anti-mouse lgG (Thermo Fisher Scientific, A-11029, A-11031; 1:1000 for staining), and DAPI (4′, 6-diamidino-2-phenylindole dihydrochloride, Sigma D-8417, 1:8000) were used to identify the nuclei. Fluorescence images were captured with an inverted fluorescence microscope (Olympus FSX100, Olympus Corp., Tokyo, Japan).

### 2.5. Immunoprecipitation (IP)

The construct of p3xFlag-HA-Myc-Nrg1 type III was transfected into HEK293 cells. Before Pierce Protein A/G Magnetic Beads (Cat. # 88803, Thermo Fisher Scientific) were mixed with the IP supernatant, the cell treatments are shown in Figure 2B. Briefly, the IP antibody was anti-Flag (Cat. # F1804, mouse, 1:1000, Sigma Aldrich), and the immunoblot antibody was anti-HA (Cat. # PRB-101P, Rabbit, 1:1000, Biolegend, San Diego, CA, USA). The input sample (90 μL) was mixed with 4× SSB (SDS-PAGE sample loading buffer, 30 μL). After IP, 20 μL IP buffer (1% Triton X-100, 150 mM NaCl, 50 mM pH 8.0 Tris, 0.5% Na-deoxycholate, 1% PMSF, and protease inhibitor cocktail) and 20 μL 2× SSB were added to elute the protein by boiling the beads for 10 min and were mixed with the IP sample. A total of 20 μL samples were subjected for each lane to SDS-PAGE, followed by WB analysis.

### 2.6. Analysis of the Images

Three replicates were performed for each experiment. Using ImageJ, we quantified the fluorescent area and intended density and analyzed the distribution trace of the images from the immunogenicity assay.

## 3. Results

The full length of Nrg1 type III is two bands in Western blotting, 140 kD (mature) and 110 kD (immature), separately (indicated by the red and green arrow in Figure 1B) [28,29,30]. The Nrg1 type III ectodomain can be cleaved by three sheddases [29,30,31,32] in the C-terminal to the EGF domain, including BACE1 (amino acid residues of shedding sites, ARSs: 283 and 285), ADAM10 (ARSs: 285 and 286), and ADAM17 (ARS: 293). 

The N-terminal processing of Nrg1 type III on the cell membrane was tested after the construct (p3xFlag-Myc-Nrg1 type III) was transfected into the N2a cell. The negative control selected the internalized Myc tag of the construct (Figure 1C). To validate the method, we added the anti-Myc antibody before (np) and after (p) the cell permeabilization, and the negative result from Myc (np) indicated that the inside part could not be detected before permeabilization (Figure 1C). A nonspecific signal of green fluorescent was also found in the inner part of the cell in the lower-panel figures, which may be caused by a dead cell with a high-permeability cell membrane. The Flag (np) staining signal (Figure 1D) suggested that part of the N-terminal of Nrg1 type III localized outside the cell due to a single-pass transmembrane. After permeabilization, antibodies to Myc (p) or Flag (p) showed strong fluorescence. The rate of the anti-Flag staining area (7.6%) and integrated density (IntDen, 12.7%) from the outside to the inner side of the cells (Figure 1E) implied that about 10% Nrg1 type III omitted the N-terminal transmembrane helix, with the N-terminal positioning outside of the membrane.

Moreover, the results from PI co-staining with Flag confirmed the N-terminal of Nrg1 type III localizing outside the cell membrane (Figure 2C,D). Before permeabilization, the anti-Flag staining signal could be found outside the cell membrane without PI-positive staining in the same cell (Figure 2A,C). Unsurprisingly, a strong anti-Flag staining signal was localizing outside the cell membrane, while the co-staining was performed after permeabilization (Figure 2B,D). The schematic model of the N-terminal distribution of Nrg1 type III on the cell membrane is shown in Figure 1F. The primary form of Nrg1 type III was a double-pass transmembrane, and the novel configuration is a single-pass transmembrane.

To confirm the outside localization of the Nrg1 type III N-terminal on the cell membrane, IP on the whole cell was executed via transfecting a new construct of Nrg1 type III (p3xFlag-HA-Myc-Nrg1 type III, as shown in Figure 3A) to Hek293 cells (Figure 3B). Conducting IP with anti-Flag, the N-terminal tag in the construct could enrich the full length of Nrg1 type III (indicated by the green and red arrow) and the fragment after cleavage (indicated by the black arrow), as shown in Figure 3C. There was a reversible binding between the IP antibody (anti-Flag) and the antigen (Flag-tag) from the novel single-pass transmembrane form of Nrg1 type III (full length) anchoring on the whole cell membrane, which potentially impacted the result. The wash step removed the unbound antibody. Then, after lysing the cells, the IP antibody may change to bind the antigen from the double-pass transmembrane form of Nrg1 type III, including its full length and N-terminal fragment. The bands indicated by arrows (red, green, and black) in the IP lane were attributed to the interaction in the first step, incubating the IP antibody with the cells.

The bands’ intensity in the IP lane was much lower than that in the corresponding input lane, although the IP volume was much more than the input volume before the IP (Figure 3B), which may also imply that the outside localization of Nrg1 type III N-terminal was a minor form on the membrane. In addition, the mature form (140 kD) was much more than the immature form (110 kD), whose relative abundance was about four times (Figure 3C,D).

**Figure 4:**
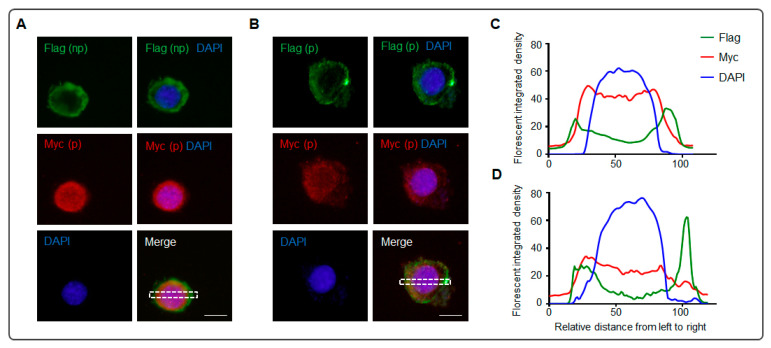
The outside distribution of Nrg1 type III on the cell membrane can be enhanced. (**A**,**B**) Representative images of an N2a cell expressing the Nrg1 type III construct (p3xFlag-Myc-Nrg1 type III) in the immunocytochemistry. The fluorescent intensity in the dash box was analyzed by ImageJ. Cell culture was supplemented with ADAM inhibitor GM6001, BACE1 inhibitor Ⅳ, and PMA (Phorbol 12-myristate 13-acetate), decreasing cleavage and promoting protein secretion, separately. (np) and (p) indicate the staining before or after cell permeabilization, separately. Anti-Flag (np) staining before the permeabilization of the cell was co-stained with anti-Myc (p) in panel (**A**). Anti-Flag (p) and anti-Myc (p) staining are shown in panel (**B**). (**C**) Analyzing the fluorescent intensity in the dash box of the panel (**A**) using ImageJ. (**D**) Analyzing the fluorescent intensity in the dash box of the panel (**B**) using ImageJ. The relative staining integrated density is shown using GraphPad Prism 6 (scale bar = 5 μm).

To enhance Nrg1 type III N-terminal localization on the outside of the cell membrane, PMA (Phorbol 12-myristate 13-acetate) was used to promote membrane protein secretion, and two cleavage inhibitors (ADAM inhibitor GM6001 and BACE1 inhibitor IV) [29,32] restrained the fragment production of Nrg1 type III. After the Nrg1 type III with both the Flag tag and Myc tag (p3xFlag-Myc-Nrg1 type III) were transfected into N2a cells, as shown in Figure 4, anti-Flag immunofluorescent staining was executed before (np, Figure 4A,C) and after (p, Figure 4B,D) permeabilization, accompanied by anti-Myc staining after permeabilization. Image J was used to analyze the distribution of the three signals (green, red, and blue) (Figure 4C,D). Green fluorescence represents the N-terminal of the Nrg1 type III, and red fluorescence represents the intracellular portion (Figure 4). The green fluorescence is around the red fluorescence, with little overlap (Figure 4C) or some overlap (left part in Figure 4D), while the green fluorescent peak is localized outside of the red fluorescent peak (Figure 4C,D). The green fluorescence of the cell before permeabilization (np) has a similar curve with the cell after permeabilization (p), confirming the function of PMA and that the N-terminal of Nrg1 type III localized the outside of the cell. The fluorescent density responding to the Flag is three times greater in the right part of panel D than in panel C, as a result of the selected area containing a highlighting signal on the right side of the rectangle in Figure 4B. The highlighted green signal in Figure 4B is the abnormal aggregation of the Nrg1 type III N-terminal, which may be due to the overexpression of the transfected constructs.

## 4. Discussion

Although Nrg1 type III encodes only 700 amino acids, its full length is considered to have two forms in WB, mature (140 kD) and immature (110 kD), separately (Figure 1B and Figure 3C) [28,29,30]. The full-length forms may be derived from the differences in the degree of modification. In Figure 1, the mature state was much less than the immature form of the full length, but the mature form became dominant in the IP on the whole-cell level (Figure 3C,D), which may be owing to the cold serum-free condition of the cell culture over the last two hours (Figure 3B). Moreover, the cleavage fragments (about 76 kD and 60 kD) in the WB suggested that the mature form was preferred to the substrate of the sheddases (Figure 1B and Figure 3C). The minor Nrg1 type III N-terminal localized outside the cell (Figure 1D and Figure 2C,D). After PMA promoted the protein secretion, and the sheddase inhibitors retained the cleavage, Nrg1 type III N-terminal localization on the outside of the cell membrane was enhanced (Figure 4A).

N-Terminal Processing of Nrg1 Type III

The cytoplasmic domain of Nrg1 type III is dispensable. Nrg1 type III can be cleaved by several sheddases, such as ADAM10, ADAM17, and BACE1, to release the EGF-like domain from the C-terminal [29,30,31,32,37]. In addition, additional cleavage sites for BACE1 and ADAM17 are found in the N-terminal to the EGF-like domain [29], which may lead to the secretion of the EGF-like domain from Nrg1 type III accompanying the cleavage of the C-terminal to the domain. Intriguingly, Nrg1 type III is also an intramembrane proteolysis substrate that can be recognized and cleaved by intramembrane-cleaving proteases [30]. The soluble EGF-like domain has been proven to stimulate ErbB signaling in a culture assay and has rescued hypomyelination due to a lack of BACE1 in a zebrafish mutant [29]. Moreover, one of the schizophrenia-associated mutations of Nrg1 type III is located in this region [30].

Most Nrg1 isoforms contain a single-pass TMD, and after proteolytic processing, they release soluble N-terminal moieties containing the EGF-signaling domain [28]. The soluble Nrg1, identified initially as GGF (glial growth factor), promotes radial neuronal migration and stimulates radial glia formation [1]. All the Nrg1 isoforms share a similar EGF-like domain in the extracellular, and Nrg1 type III includes double-pass TMDs with the EGF domain in the extracellular loop [15]. CRD in the Nrg1 type III N-terminal cytoplasmic region overlaps with the transmembrane helix, and the ectodomain can be cleaved in the C-terminal to the EGF domain [29,30,31,32]. Therefore, Nrg1 type III endows its acting as a juxtracrine signal.

This study found that a novel form of Nrg1 type III via N-terminal processing contained a single-pass TMD similar to Nrg1’s other isoforms on the membrane, which may be a consequence of gene overexpression in vitro or may exhibit functions in a novel manner in vivo. After cleavage at the C-terminal of EGF by the sheddases in Nrg1 type III [29,30,31,32], a double-pass transmembrane form of Nrg1 type III generated membrane-anchored mature Nrg1 function in a cell-contact manner. Meanwhile, a single-pass transmembrane form of Nrg1 type III released the soluble Nrg1 type III, function in a paracrine signaling pathway.

Although axonal myelination control is remarkably dissimilar in the CNS and PNS, the myelinated neurons express Nrg1 Type III [38]. NRG1 type III is abundant in the brain, such as in the piriform cortex, layer 5 of the cerebral cortex, the thalamus reticular nucleus, and the hippocampus [5,38]. In the PNS, Nrg1 type III is the most prominent isoform in motoneurons and sensory neurons, and axonal Nrg1 type III controls Schwann cell development and myelination. Indeed, the anchored Nrg1 Type III on the axonal surface provides axon size information to the associated Schwann cells [39]. Therefore, the soluble Nrg1 type III from the novel N-terminal processing may diffuse to stimulate Schwann cell or oligodendrocyte development to some extent.

More intriguingly, N-terminus 296 amino acid residues of Nrg1 type III were soluble, composed of both the CRD and EGF domains (Figure 1B) [40,41,42]. A small amount of Nrg1 type III was released into the culture medium, though it mainly anchored on the cell surface [28,29]. The C-terminal TMD of Nrg1 type III remained effective, so the original N-terminal TMD deficiency did not destroy its tight association with the cell membrane. Some potential modifications in the N-terminal TMD may affect the alpha helix, such as phosphorylation, glycosylation, and even the disulfide linkage in the CRD (Figure 1E). Admittedly, the mechanism needs appropriate assays in order to verify it.

## 5. Conclusions

Nrg1 type III N-terminal processing is a complicated course. The primary form of Nrg1 type III on the cell membrane is the double-pass transmembrane, and the single-pass transmembrane is a novel form of Nrg1 type III via N-terminal processing (Figure 1E).

## Data Availability

Data sharing does not apply to this article, as no datasets were generated or analyzed during the current study.

## Supplementary Materials

The following supporting information can be downloaded at: https://www.mdpi.com/article/10.3390/biom13121756/s1, File S1: Original Western Blotting Figures of Figure 1; File S2: Original Western Blotting Figures of Figure 3.

## Abbreviations

ALS, amyotrophic lateral sclerosis; ARSs, amino acid residues of shedding sites; CRD, cysteine-rich domain; CNS, central nervous system; DAPI, 4′, 6-diamidino-2-phenylindole dihydrochloride; EGF, epidermal growth factor; FBS, fetal bovine serum; IP, immunoprecipitation; Fig, Figure; Nrg1, neuregulin 1; PKA, protein kinase A; PI, propidium iodide; PMSF, phenyl-methane sulfonyl fluoride; PNS, peripheral nervous system; SDS-PAGE, sodium dodecyl sulfate–polyacrylamide gel electrophoresis; TMD, transmembrane domain; WB, Western blotting.
