# Supplementary figures and images for: A Novel Form of Neuregulin 1 Type III Caused by N-Terminal Processing
Source: Biomolecules. 2023 Dec 7;13(12):1756. doi: 10.3390/biom13121756 (PMC10741733; doi:10.3390/biom13121756)

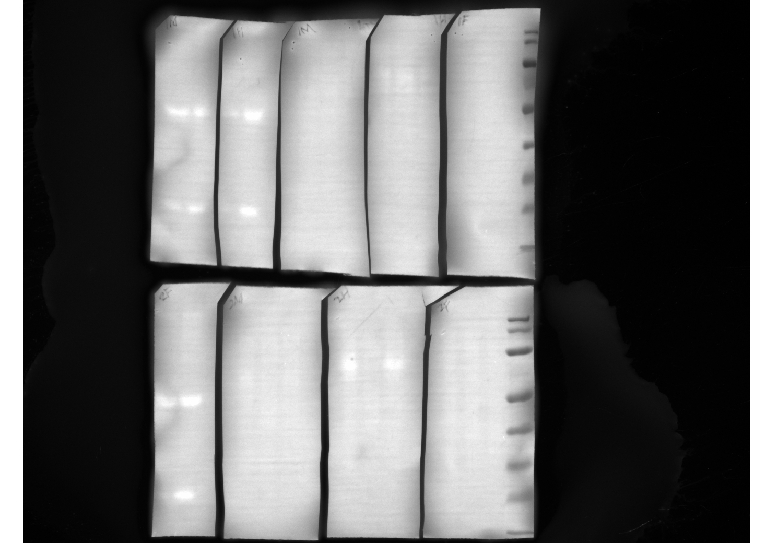

Supplement: Supplementary file 1 [file biomolecules-13-01756-s001.zip › File S1/08-11 16 h 26 m 00.jpg]

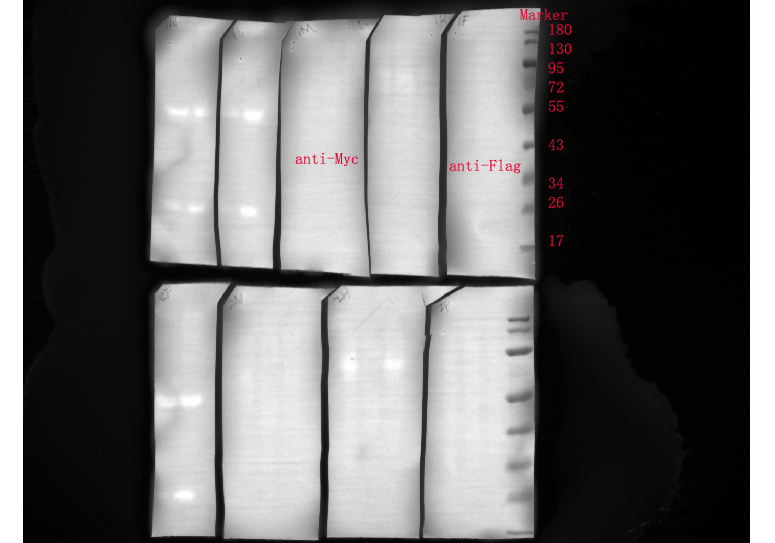

Supplement: Supplementary file 1 [file biomolecules-13-01756-s001.zip › File S1/08-11 16 h 26 m00 -sw.tif]

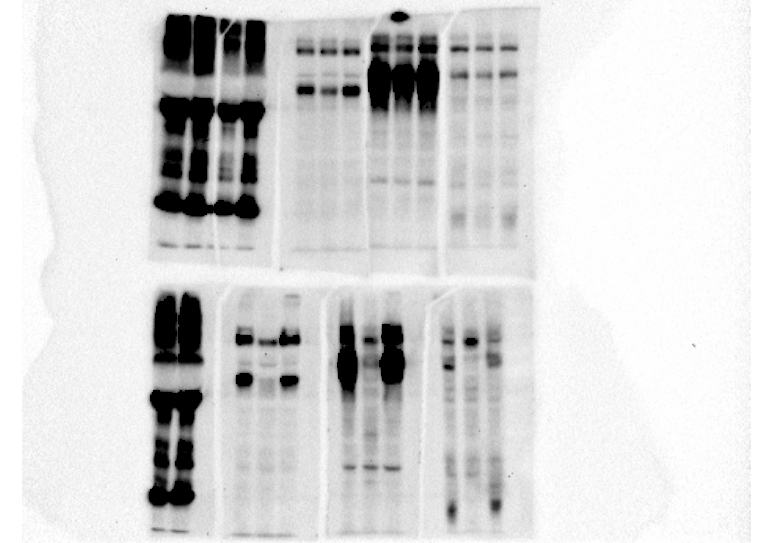

Supplement: Supplementary file 1 [file biomolecules-13-01756-s001.zip › File S1/Exposure_331.6sec.jpg]

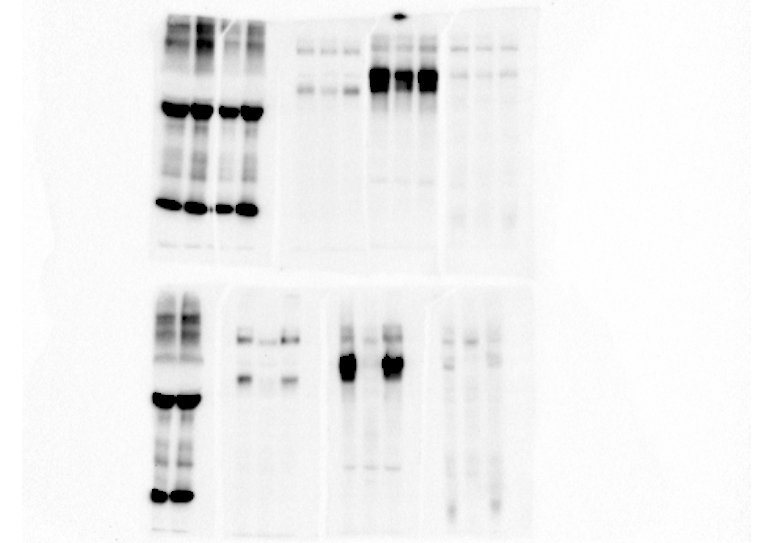

Supplement: Supplementary file 1 [file biomolecules-13-01756-s001.zip › File S1/Exposure_74.5sec-sw.jpg]

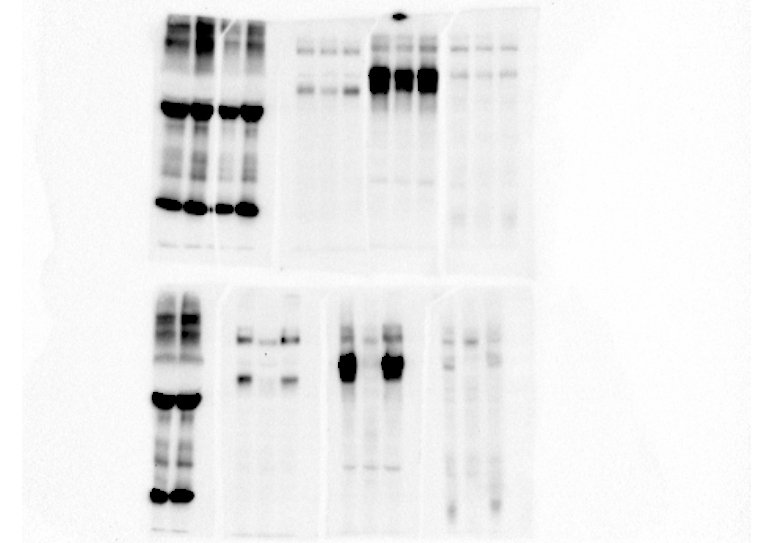

Supplement: Supplementary file 1 [file biomolecules-13-01756-s001.zip › File S1/Exposure_99.0sec.jpg]

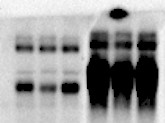

Supplement: Supplementary file 1 [file biomolecules-13-01756-s001.zip › File S1/high.jpg]

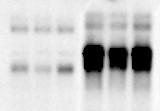

Supplement: Supplementary file 1 [file biomolecules-13-01756-s001.zip › File S1/middle.jpg]

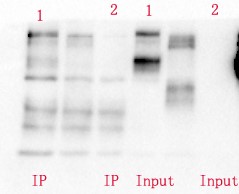

Supplement: Supplementary file 1 [file biomolecules-13-01756-s001.zip › File S2/001.jpg]

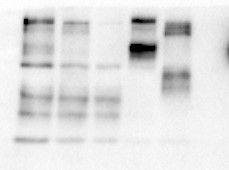

Supplement: Supplementary file 1 [file biomolecules-13-01756-s001.zip › File S2/002.jpg]

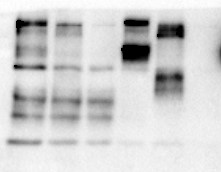

Supplement: Supplementary file 1 [file biomolecules-13-01756-s001.zip › File S2/003.jpg]

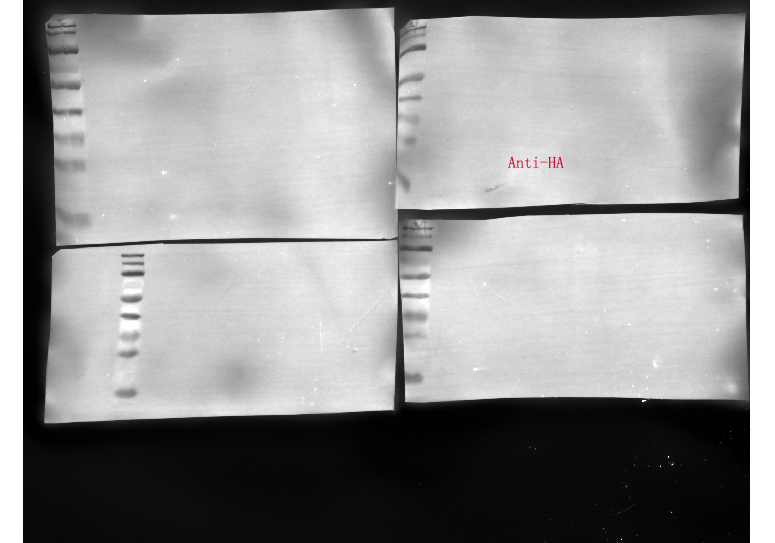

Supplement: Supplementary file 1 [file biomolecules-13-01756-s001.zip › File S2/02-23 15 h 48 m - backup.jpg]

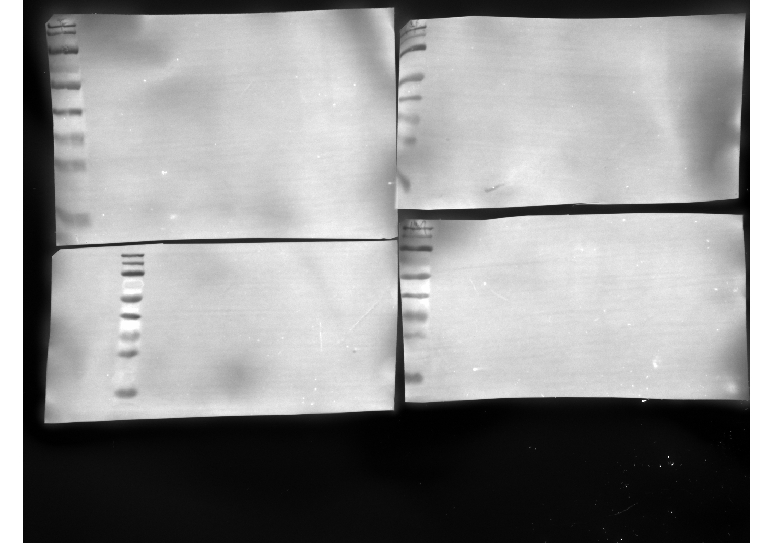

Supplement: Supplementary file 1 [file biomolecules-13-01756-s001.zip › File S2/02-23 15 h 48 m.jpg]

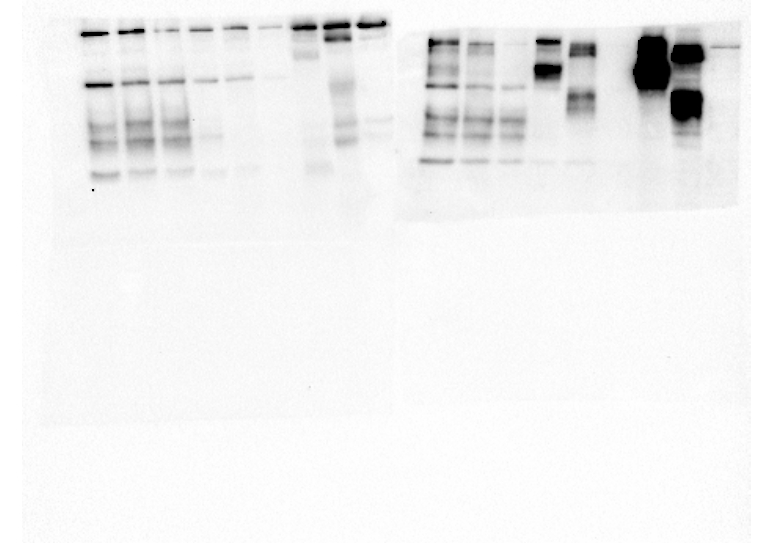

Supplement: Supplementary file 1 [file biomolecules-13-01756-s001.zip › File S2/Exposure_111.2sec.jpg]

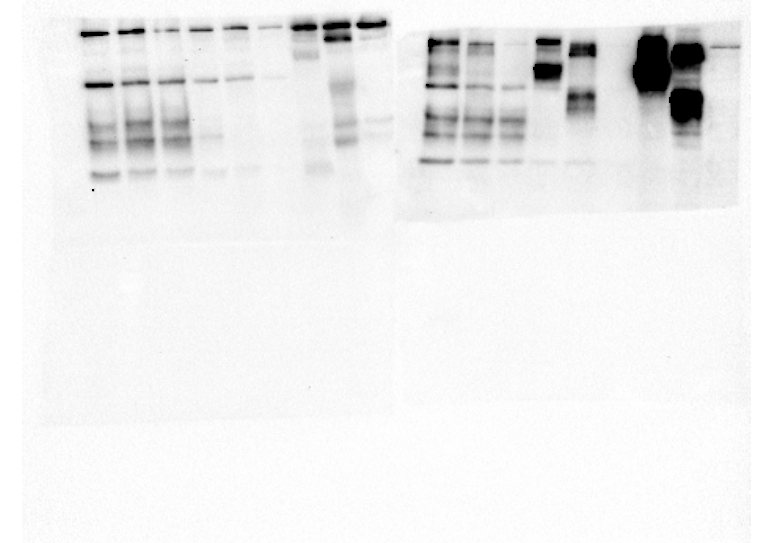

Supplement: Supplementary file 1 [file biomolecules-13-01756-s001.zip › File S2/Exposure_141.8sec.jpg]

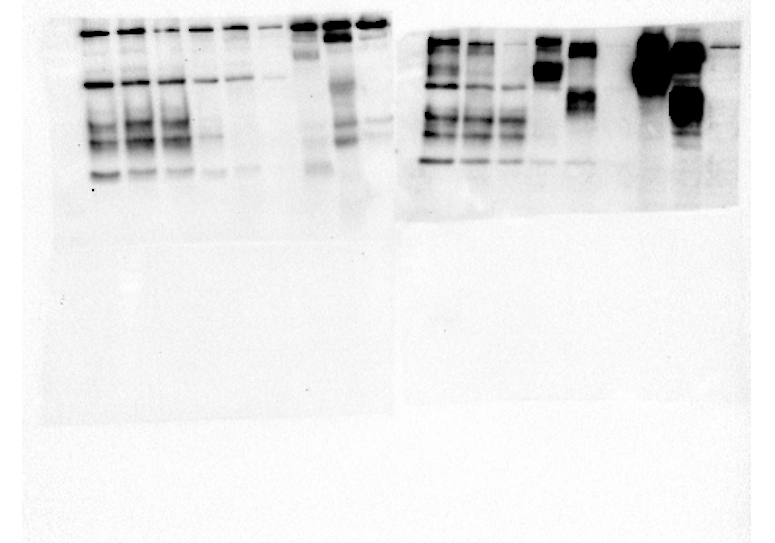

Supplement: Supplementary file 1 [file biomolecules-13-01756-s001.zip › File S2/Exposure_221.4sec.jpg]

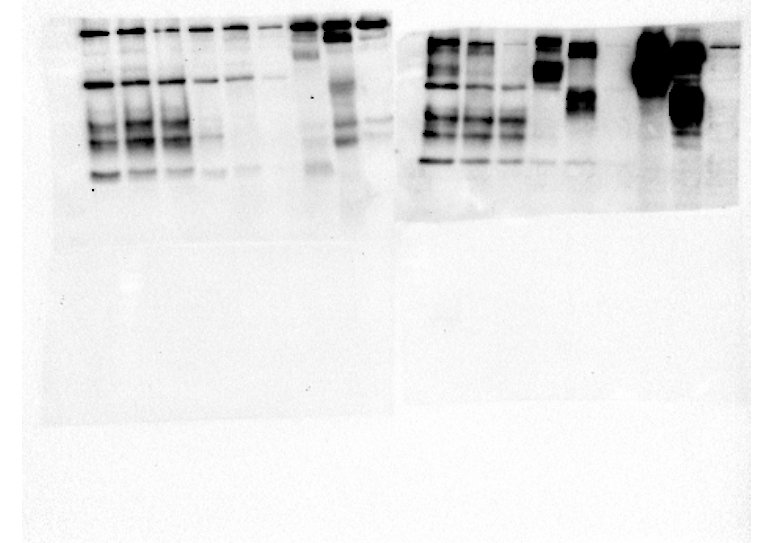

Supplement: Supplementary file 1 [file biomolecules-13-01756-s001.zip › File S2/Exposure_258.1sec.jpg]

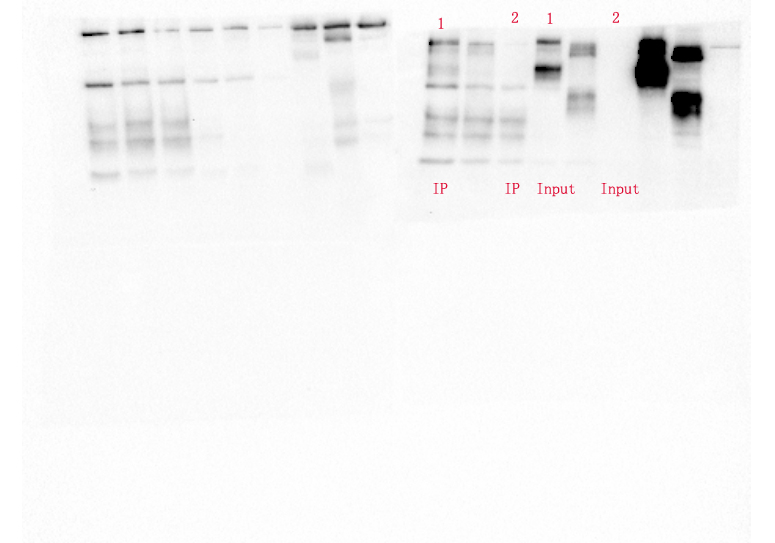

Supplement: Supplementary file 1 [file biomolecules-13-01756-s001.zip › File S2/Exposure_62.2sec - backup.jpg]

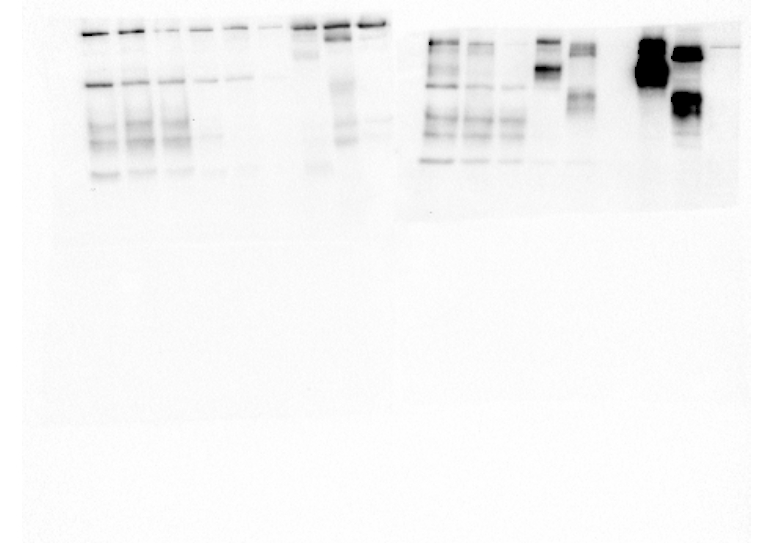

Supplement: Supplementary file 1 [file biomolecules-13-01756-s001.zip › File S2/Exposure_62.2sec.jpg]

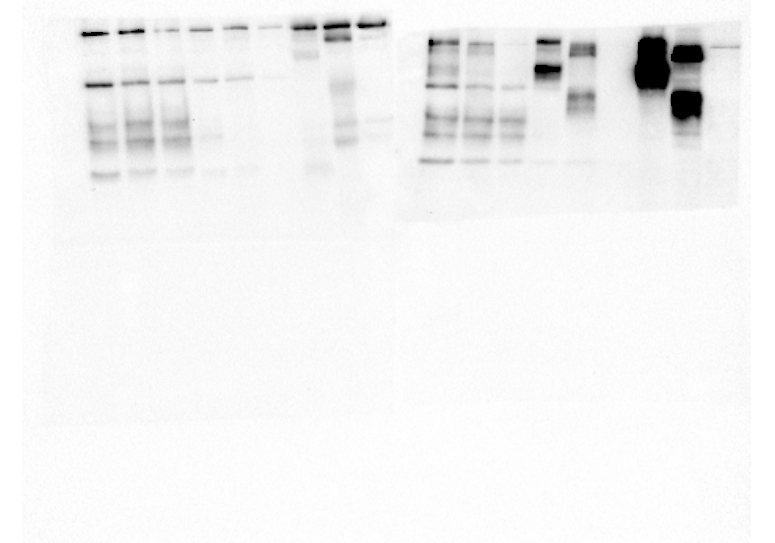

Supplement: Supplementary file 1 [file biomolecules-13-01756-s001.zip › File S2/Exposure_86.7sec.jpg]
